# Comparative Evaluation of Intraperitoneal Instillation of Bupivacaine With Magnesium Sulfate Versus Bupivacaine With Dexmedetomidine for Postoperative Relief After Laparoscopic Cholecystectomy

**DOI:** 10.7759/cureus.102057

**Published:** 2026-01-22

**Authors:** Aditi R Singh, Navneet Gupta, Jyothi Chaudhary, Subhash Dahiya, Garima Luthra, Namrata Kaka

**Affiliations:** 1 Anesthesiology, Critical Care, and Pain Medicine, Lala Lajpat Rai Memorial (LLRM) Medical College, Meerut, IND; 2 Anesthesiology, Lala Lajpat Rai Memorial (LLRM) Medical College, Meerut, IND

**Keywords:** intraperitoneal instillation, laparoscopic cholecystectomy, postoperative pain, rescue analgesia, visual analogue scale (vas)

## Abstract

Background: Laparoscopic cholecystectomy (LC) is the most common surgical treatment for cholelithiasis and offers advantages over open surgery. However, postoperative pain remains a significant concern, arising from both somatic and visceral components. Intraperitoneal instillation of local anesthetics with adjuvants has been explored as a simple and effective method of pain control.

Objective: This study compared intraperitoneal bupivacaine with magnesium sulfate versus bupivacaine with dexmedetomidine for postoperative pain relief in LC. Pain assessed using the Visual Analog Scale (VAS), analgesia duration, rescue analgesic use, and hemodynamic stability were evaluated.

Materials and methods: A prospective, randomized, double-blind clinical study was conducted on 116 American Society of Anesthesiologists I and II patients undergoing elective LC. Patients were divided into two groups: Group A received intraperitoneal bupivacaine with magnesium sulfate, and Group B received intraperitoneal bupivacaine with dexmedetomidine. Pain scores, analgesic duration, rescue analgesic requirement, and hemodynamic parameters were compared between the groups.

Results: Both groups were comparable with respect to demographic variables. Group A showed significantly lower VAS scores at two, four, and six hours postoperatively (p < 0.001), indicating better early pain control. The duration of analgesia and total rescue analgesic consumption were comparable between groups. Hemodynamic parameters were stable, though Group B exhibited lower heart rate and blood pressure at some time intervals, consistent with the sympatholytic effect of dexmedetomidine. Both regimens were well tolerated, with minimal side effects.

Conclusions: Intraperitoneal instillation of bupivacaine with magnesium sulfate provides superior early postoperative analgesia compared to bupivacaine with dexmedetomidine, while both combinations are safe and effective. Magnesium sulfate is a cost-effective, readily available adjuvant that may be considered a better alternative in multimodal analgesia for LC.

## Introduction

Laparoscopic cholecystectomy (LC) derives from the Greek *lapára* (flank) and *skopeō* (to view), whereas “cholecystectomy” comes from cholecyst (gallbladder) and ectomy (removal) [1]. Carl Langebuch performed the first cholecystectomy in 1882, and Erich Mühe introduced the laparoscopic approach in 1985 [2]. Since its introduction, LC has become the gold standard for managing symptomatic gallstone disease, offering advantages such as reduced postoperative pain, shorter hospital stays, faster recovery, and improved cosmetic outcomes.

The minimally invasive nature of LC has revolutionized surgical practice. Patients often prefer this approach because it facilitates early mobilization and allows day-care management in many cases, making LC one of the most frequently performed elective surgeries worldwide. Compared with open procedures, LC is associated with smaller incisions, minimal blood loss, faster recovery, and better postoperative comfort.

Despite these advantages, LC is still associated with significant postoperative pain. This pain, largely visceral in nature, arises from diaphragmatic irritation, peritoneal inflammation, and residual intraperitoneal carbon dioxide used during pneumoperitoneum [3]. Effective postoperative pain management is crucial because inadequate control may delay recovery, contribute to chronic pain, impair ventilation, increase sympathetic activity, and prolong hospitalization [4].

Several modalities have been explored to manage pain after LC, including systemic analgesics (e.g., nonsteroidal anti-inflammatory drugs and opioids), regional anesthesia techniques such as epidural or transversus abdominis plane blocks, and local anesthetic infiltration [5]. Among these, intraperitoneal instillation of local anesthetics has gained popularity because it is simple, safe, and effective. Bupivacaine, a long-acting amide local anesthetic, is frequently used for this purpose. The addition of adjuvants such as dexmedetomidine and magnesium sulfate can further enhance analgesic efficacy [6,7].

Dexmedetomidine, a highly selective α2-adrenergic agonist, provides both sedative and analgesic effects and has been associated with reduced opioid requirements without causing respiratory depression [8]. Magnesium sulfate is a physiological N-methyl-D-aspartate receptor antagonist and calcium-channel blocker that reduces central sensitization and thereby exerts analgesic effects [9].

Given these potential benefits, the present study aimed to compare the postoperative analgesic efficacy of intraperitoneal bupivacaine combined with either dexmedetomidine or magnesium sulfate in patients undergoing LC.

## Materials and methods

This prospective, randomized, double-blind study was conducted at Sardar Vallabh Bhai Patel Hospital, an affiliated institution of Lala Lajpat Rai Memorial Medical College, Meerut. A total of 116 patients (American Society of Anesthesiologists (ASA) physical status I-II [10], aged 18-65 years, either sex) scheduled for elective LC under general anesthesia were enrolled. Ethical clearance was obtained from the Institutional Ethics Committee of Lala Lajpat Rai Memorial Medical College (approval number: SC-1/024/4696, approval date: June 22, 2024) prior to commencement, and written informed consent was obtained from all participants. The study was registered in the Clinical Trials Registry of India (CTRI/2024/10/074565).

Study design and sample size

**Figure 1:**
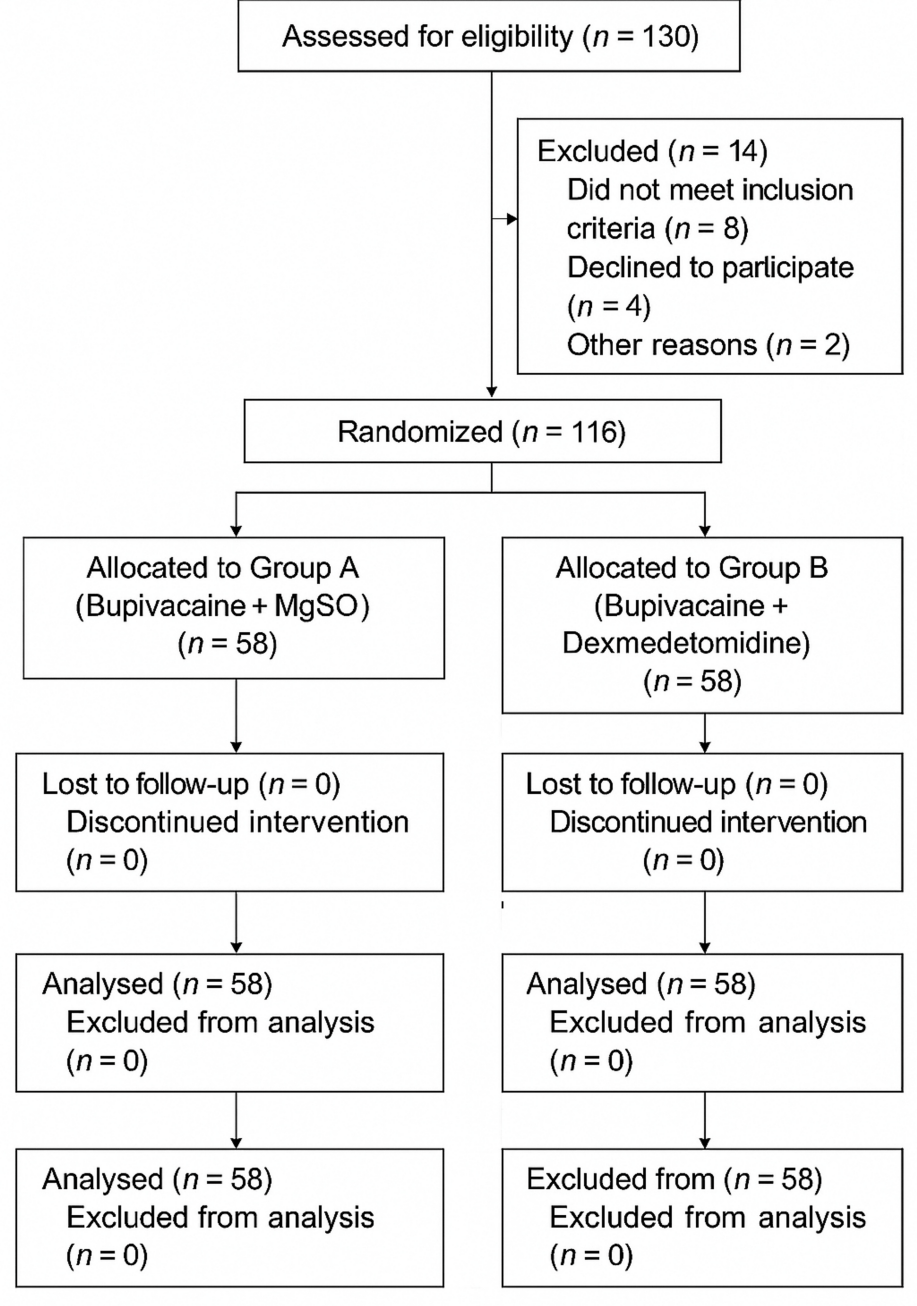
CONSORT 2010 flow diagram A total of 130 patients were assessed for eligibility, 14 were excluded, and 116 were randomized equally into two groups (58 each). All participants received the allocated intervention, completed follow-up, and were included in the final analysis.

The study followed a prospective, randomized design. Sample size calculation, performed using Power Analysis and Sample Size version 23 (NCSS, LLC, Kaysville, UT, USA), was based on an anticipated 25% difference in postoperative pain score reduction between the two groups, with a two-sided 95% confidence interval and 80% power. This yielded a requirement of 58 patients per group (n = 116). Randomization was achieved using the sealed-envelope technique. An anesthesiologist performed drug preparation and administration, while an independent, blinded observer carried out postoperative assessments to ensure objectivity (Figure 1).

Group allocation

The patients were randomized into two groups, with 58 participants in each group. Group A received a combination of 20 mL of 0.25% bupivacaine and 50 mg/kg magnesium sulfate, diluted to a total volume of 30 mL. Group B received 20 mL of 0.25% bupivacaine combined with 1 mcg/kg dexmedetomidine, diluted to a total volume of 30 mL. At the end of the surgical dissection, the study drug was administered intraperitoneally to all patients.

Inclusion and exclusion criteria

The study included patients of either sex between the ages of 18 and 65 years who had an ASA physical status of I or II. All participants were scheduled to undergo elective LC and provided written informed consent prior to their inclusion in the study.

Patients with an ASA physical status of III or IV, known hypersensitivity to local anesthetics, or a history of asthma, chronic lung disease, cardiac disease, or renal failure, or those undergoing dialysis were excluded from the study. Pregnant patients, those who refused to participate, and cases where the procedure was converted to open cholecystectomy were also excluded. Additionally, individuals with psychiatric illness or coagulopathy were not included in the study.

Preoperative preparation

All patients underwent a pre-anesthetic evaluation, including weight, airway assessment, and cardiovascular, respiratory, and neurological evaluation. Baseline investigations included hemoglobin, total leukocyte count, platelet count, and prothrombin time. Patients were kept nil per os for six hours before surgery. A 10-cm Visual Analog Scale (VAS) was explained for postoperative pain assessment (0 = no pain, 10 = worst pain imaginable) [11].

Intraoperative management

Standard monitoring included heart rate (HR), systolic blood pressure (SBP), diastolic blood pressure (DBP), respiratory rate (RR), oxygen saturation (SpO₂), and temperature. Intravenous access was secured, and crystalloids were administered at 20 mL/kg/h.

Premedication consisted of IV midazolam 0.05 mg/kg. General anesthesia was induced with propofol 2 mg/kg, fentanyl 1-2 mcg/kg, and vecuronium (loading dose 0.08-0.1 mg/kg; maintenance 0.01-0.02 mg/kg). Patients were intubated following adequate relaxation. Anesthesia was maintained with isoflurane in an oxygen-air mixture, with ventilation adjusted to maintain EtCO₂ between 34 and 38 mmHg. Supplemental fentanyl 0.5 mcg/kg IV was administered as needed.

LC was performed using a standard three-port technique. Pneumoperitoneum was established with carbon dioxide, maintaining intra-abdominal pressure at 10-12 mmHg. Port-site infiltration was performed with 4 mL of 1% lignocaine (maximum dose 3 mg/kg).

At the conclusion of surgery, the study drug was instilled intraperitoneally after peritoneal irrigation and suction. The patient was placed in a right Trendelenburg position for 5 minutes to facilitate drug dispersion. Neuromuscular blockade was reversed with neostigmine (0.05-0.08 mg/kg) and glycopyrrolate (0.005-0.01 mg/kg). Patients were extubated and transferred to the post-anesthesia care unit (PACU).

Postoperative assessment

Pain was assessed using the VAS at PACU admission (time 0) and at two, four, six, 12, and 24 hours postoperatively, both for abdominal and shoulder tip pain. Sedation was evaluated using the Ramsay Sedation Scale.

Validity and reliability of the pain assessment tool

Postoperative pain was assessed using the VAS, a widely validated and widely used tool for measuring acute postoperative pain. The VAS consists of a 10-cm horizontal line ranging from 0 (no pain) to 10 (worst imaginable pain). It has been shown to possess excellent construct validity and sensitivity in detecting changes in pain intensity across various surgical populations.

The scale demonstrates high reliability and repeatability, with previous studies reporting strong test-retest reliability (intraclass correlation coefficient >0.90) and good internal consistency. Its validity in postoperative pain assessment has been well established, making it a standard outcome measure in analgesic trials, including laparoscopic procedures. The simplicity, reproducibility, and patient acceptability of VAS make it an appropriate and reliable tool for assessing postoperative pain in the present study.

Rescue analgesia was administered when the VAS exceeded 4 or upon patient request. Intravenous tramadol (1.5 mg/kg) was the first-line agent, with diclofenac (1 mg/kg IV) given if pain persisted after 30 minutes. Maximum allowable doses were 4 mg/kg for tramadol (minimum interval four hours) and 3 mg/kg/24 h for diclofenac (minimum interval six hours). Episodes of postoperative nausea and vomiting (PONV) were treated with ondansetron 4 mg IV.

Adverse events were closely monitored for 24 hours. Bradycardia (HR decrease ≥20% from baseline) was treated with atropine 0.02 mg/kg, tachycardia (HR increase ≥20%) with fentanyl 0.5 mcg/kg, hypotension (SBP decrease ≥20%) with crystalloids, and hypertension (SBP increase ≥20%) with fentanyl boluses.

Statistical analysis

Data were analyzed using SPSS Statistics version 20.0 (IBM Corp., Released 2011. IBM SPSS Statistics for Windows, Version 20.0. Armonk, NY: IBM Corp.). Continuous variables were expressed as mean ± SD and compared with the Student’s t-test for normally distributed data or the Mann-Whitney U test for skewed data. Categorical variables were presented as frequencies and percentages and analyzed using the chi-square test. A p-value <0.05 was considered statistically significant.

## Results

A total of 116 patients aged 18 to 65 years were enrolled and randomly allocated to two equal groups of 58 each. Group A received 20 mL of 0.25% bupivacaine combined with 50 mg/kg magnesium sulfate (total 30 mL), while Group B received 20 mL of 0.25% bupivacaine combined with 1 µg/kg dexmedetomidine (total 30 mL). All participants completed the study and were included in the final analysis (Table 1).

**Table 1:** Demographic and clinical characteristics of study participants Values are presented as mean ± SD or number (percentage) as appropriate. Comparisons between groups were performed using the Student’s t-test for continuous variables and the chi-square test for categorical variables. A p-value < 0.05 was considered statistically significant. ASA: American Society of Anesthesiologists, BP: blood pressure, SD: standard deviation, SpO₂: oxygen saturation, DBP: diastolic blood pressure, SBP: systolic blood pressure

Parameter	Group A (bupivacaine + MgSO₄, n = 58)	Group B (bupivacaine + dexmedetomidine, n = 58)	p-value
Age (years)			
≤30	25 (43.1 %)	25 (43.1 %)	
31-40	14 (24.1 %)	15 (25.9 %)	
41-50	10 (17.2 %)	13 (22.4 %)	
>50	9 (15.5 %)	5 (8.6 %)	
Mean ± SD	37.45 ± 11.9	35.53 ± 10.7	0.366
Gender			
Male	6 (10.3 %)	6 (10.3 %)	
Female	52 (89.7 %)	52 (89.7 %)	1.000
Anthropometry			
Height (cm)	158.6 ± 4.2	157.1 ± 4.0	0.060
Weight (kg)	60.5 ± 7.2	58.9 ± 9.2	0.293
BMI (kg/m²)	24.0 ± 2.5	23.7 ± 3.0	0.584
ASA grade	I = 41 (70.7 %) II = 17 (29.3 %)	I = 46 (79.3 %) II = 12 (20.7 %)	0.284
Comorbidity present	8 (13.8 %)	4 (6.9 %)	0.765
Baseline vitals			
Pulse rate (beats/min)	91.4 ± 12.1	92.5 ± 12.5	0.658
SBP (mmHg)	127.8 ± 13.4	124.9 ± 11.8	0.215
DBP (mmHg)	80.2 ± 9.0	79.0 ± 7.8	0.432
SpO₂ (%)	98.6 ± 0.5	98.7 ± 0.6	0.644

Postoperative pain assessment

**Figure 2:**
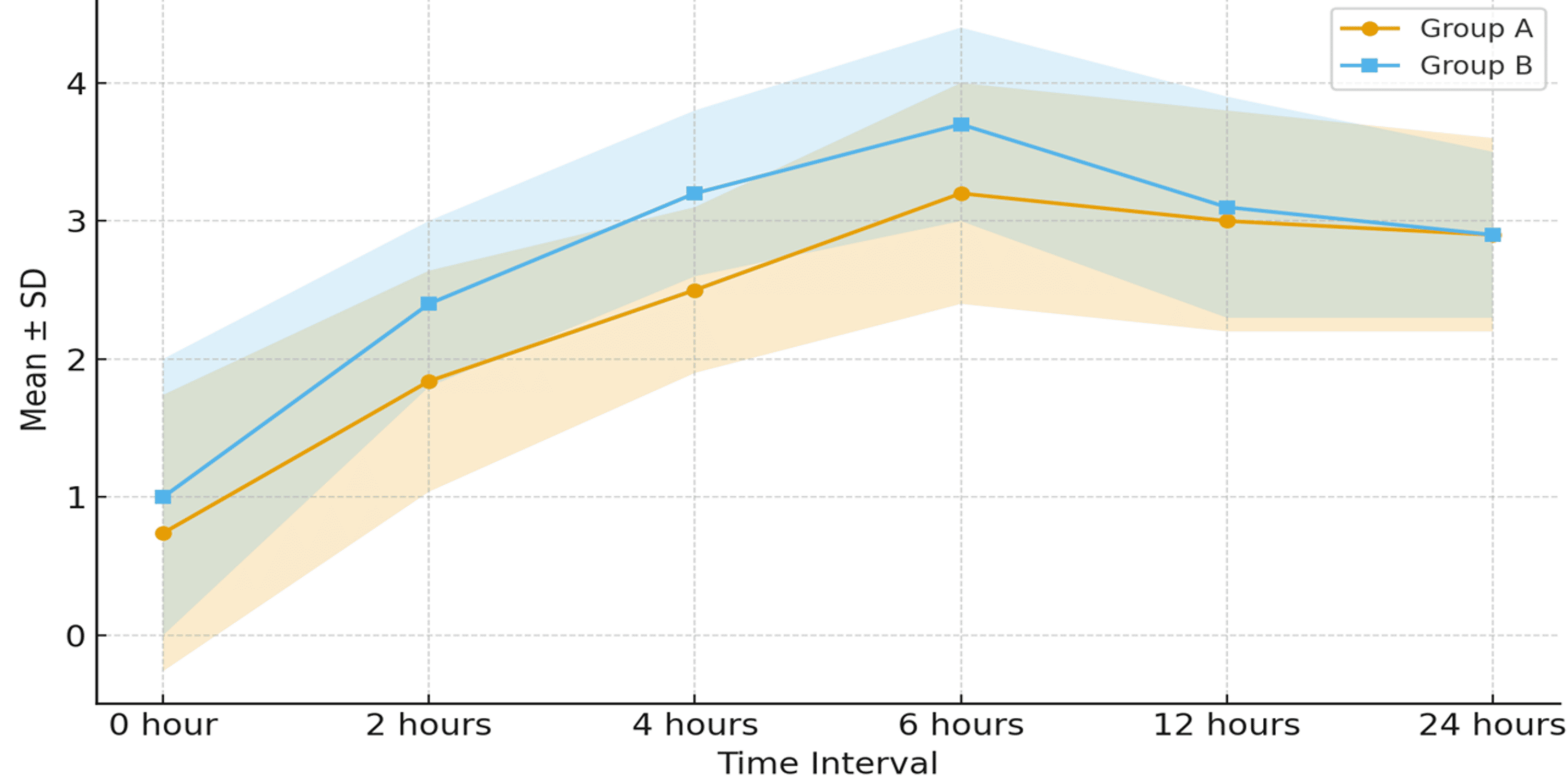
Postoperative VAS score comparison between groups Line graph showing mean VAS pain scores (0–10) at 0, 2, 4-, 6-, 12-, and 24-hours post-surgery. Group A (bupivacaine + magnesium sulfate) demonstrated significantly lower scores at four and six hours (p < 0.05), indicating superior early postoperative analgesia. VAS: Visual Analog Scale, SD: standard deviation

Pain intensity was measured using the VAS at 0, 2, 4, 6, 12, and 24 hours postoperatively. Mean VAS scores were comparable at baseline (0 hours), two hours, and 24 hours (p = 0.682). However, Group A demonstrated significantly lower pain scores at four hours (2.5 ± 0.6 vs 3.2 ± 0.6; p < 0.001) and 6 hours (3.2 ± 0.8 vs 3.7 ± 0.7; p = 0.001) than Group B, reflecting superior early postoperative analgesia. At 12 hours, the difference was not statistically significant (3.0 ± 0.8 vs 3.1 ± 0.8; p = 0.574), and pain scores remained comparable thereafter (Figure 2).

Rescue analgesia requirement

**Figure 3:**
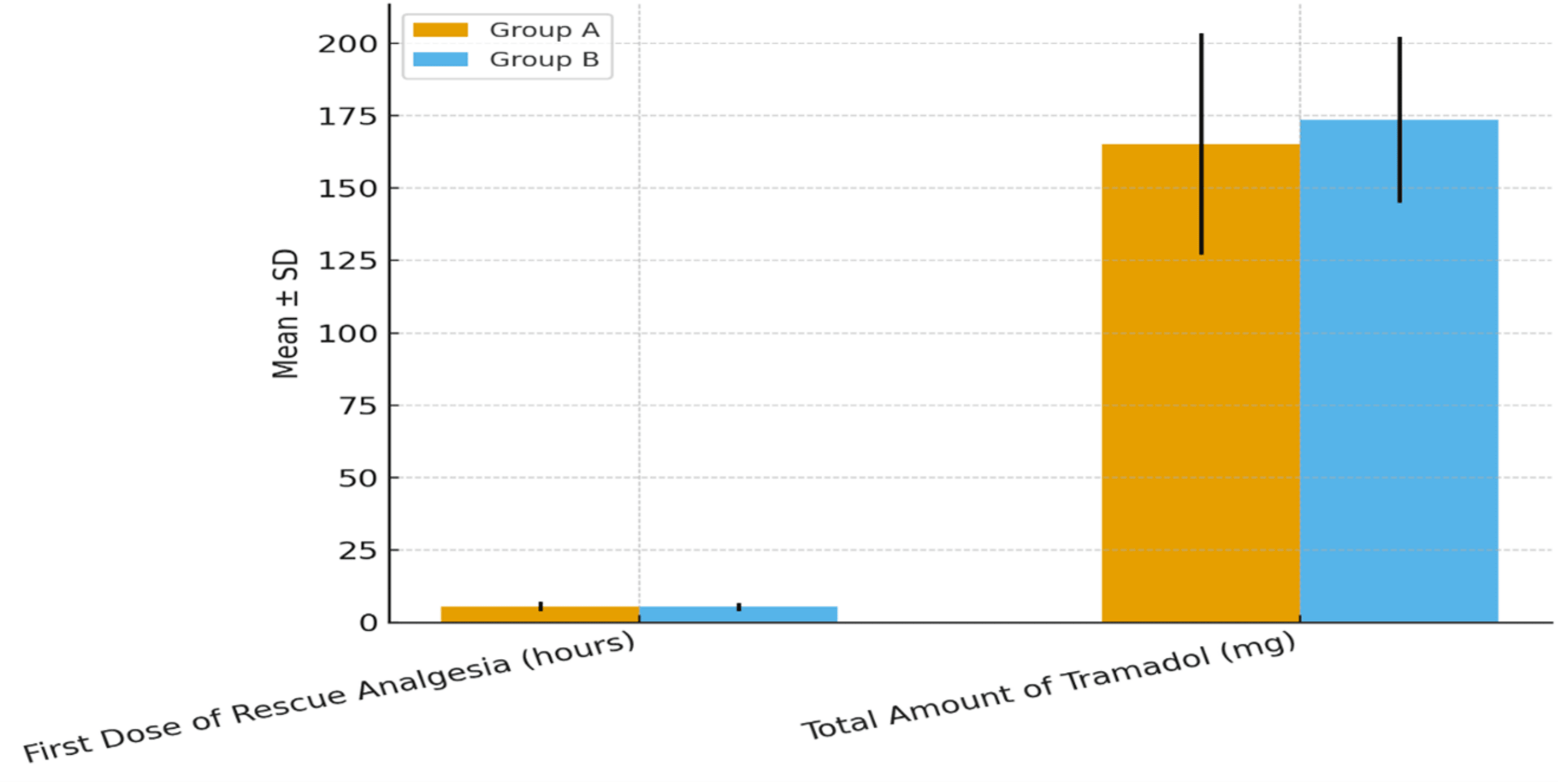
Bar chart showing the total tramadol consumption within 24 hours in both groups Bar chart showing the mean ± SD of total tramadol consumption (mg) during the first 24 postoperative hours. Group A required a mean of 165.17 ± 38.26 mg, whereas Group B required 173.53 ± 28.59 mg, with no statistically significant difference between groups (p = 0.186). SD: standard deviation

The mean time to first rescue analgesic was 5.45 ± 1.6 hours in Group A and 5.31 ± 1.3 hours in Group B (p = 0.636). The total tramadol consumption within 24 hours was 165.17 ± 38.26 mg in Group A and 173.53 ± 28.59 mg in Group B (p = 0.186), showing no statistically significant difference between groups. These findings indicate that while magnesium sulfate offered better early pain relief, the overall analgesic requirement within 24 hours remained similar (Figure 3).

Hemodynamic parameters

HR, SBP, DBP, and SpO₂ remained within physiological limits throughout the intraoperative and postoperative periods in both groups. Group B demonstrated slightly lower mean HR and blood pressure at isolated time intervals, consistent with the sympatholytic action of dexmedetomidine, but these changes were clinically insignificant.

Adverse effects

No major intra- or postoperative complications occurred. Minor adverse effects such as postoperative nausea, vomiting, and shoulder tip pain were minimal, self-limiting, and comparable between the two groups. No episodes of bradycardia, hypotension, or excessive sedation were observed in any patient.

## Discussion

The present randomized, double-blind study demonstrated that intraperitoneal instillation of bupivacaine with magnesium sulfate provided superior early postoperative analgesia compared to bupivacaine with dexmedetomidine in patients undergoing LC. Despite the minimally invasive nature of laparoscopic surgery, visceral and parietal pain resulting from peritoneal irritation, diaphragmatic stretch, and residual carbon dioxide insufflation continues to be a significant cause of postoperative discomfort. Therefore, effective analgesia remains essential for optimal recovery, early ambulation, and timely discharge.

Both groups in this study were comparable in demographic and baseline characteristics, confirming proper randomization. Significantly lower VAS scores at four and six hours in the magnesium sulfate group reflected superior early pain control, which is clinically relevant because most LC patients are discharged within 24 hours. The early analgesic advantage of magnesium sulfate may thus contribute to improved recovery profiles in day-care surgical practice.

The enhanced analgesic effect of magnesium sulfate can be attributed to its N-methyl-D-aspartate receptor antagonism and calcium-channel blocking properties, which reduce central sensitization and attenuate hyperalgesia. These mechanisms modulate spinal nociceptive transmission and complement bupivacaine's local anesthetic action. Similar results were reported by Chen et al. [12] and Ryu et al. [13], who observed decreased postoperative pain and reduced analgesic consumption when magnesium sulfate was used with local anesthetics in laparoscopic procedures. Shakya et al. [14] also demonstrated that magnesium sulfate provides better early analgesia than dexmedetomidine, although total 24-hour analgesic consumption remained comparable.

Dexmedetomidine, a highly selective α₂-adrenergic agonist, produces analgesia and sedation by inhibiting sympathetic outflow and reducing norepinephrine release, thereby decreasing nociceptive transmission. However, its slower onset and limited absorption from the peritoneal surface may explain its relatively delayed analgesic effect compared with magnesium sulfate. Praveena et al. [15] observed similar findings, showing that although dexmedetomidine enhances postoperative analgesia when combined with local anesthetics, it may not be superior to other adjuvants such as magnesium sulfate.

Rescue analgesia findings complemented the pain-score results, with comparable times to first analgesic requirement and total tramadol consumption between groups. This indicates that while magnesium sulfate offered better early relief, the overall 24-hour analgesic demand was similar, highlighting the multifactorial nature of postoperative pain perception and individual variability in analgesic response.

Hemodynamic parameters remained within physiological limits in both groups [16]. The slightly lower HR and blood pressure in the dexmedetomidine group were clinically insignificant and consistent with its known sympatholytic and vagomimetic effects. Similar hemodynamic stability was reported by Oza et al. [17], who documented minor cardiovascular changes without adverse events following intraperitoneal administration of dexmedetomidine.

Shoulder tip pain and PONV were minimal and comparable between the two groups. Comparable findings have been documented by Shukla et al. [18], Oza et al. [17], and Fares et al. [19], emphasizing the role of intraperitoneal local anesthetic instillation in reducing referred and visceral pain after laparoscopic surgery. Meticulous evacuation of residual CO₂ and thorough peritoneal lavage likely contributed to this outcome [20].

This study has certain limitations. Pain perception is subjective despite standardized evaluation using VAS. The sample size was modest, and only ASA I-II patients were included, limiting the generalizability of the findings. Furthermore, the single-center design and short 24-hour follow-up did not allow assessment of long-term outcomes or delayed complications. Measurement of serum drug concentrations could have provided additional insight into systemic absorption and pharmacodynamics of the adjuvants.

Overall, the findings suggest that intraperitoneal bupivacaine with magnesium sulfate provides superior early postoperative analgesia while maintaining hemodynamic stability and safety comparable to those of dexmedetomidine. Given its low cost, wide availability, and proven efficacy, magnesium sulfate is a practical, effective, and cost-effective adjuvant for multimodal analgesia in LC, particularly valuable in day-care and resource-limited settings.

## Conclusions

Intraperitoneal instillation of bupivacaine with adjuvants provides effective postoperative pain control following LC. In this study, the combination of 50 mg/kg magnesium sulfate with 20 mL of 0.25% bupivacaine offered superior analgesia compared with 1 µg/kg dexmedetomidine plus 20 mL of 0.25% bupivacaine, particularly during the early postoperative period at two, four, and six hours, as reflected by significantly lower VAS scores. The duration of analgesia and total analgesic consumption were comparable between the two groups.

Both regimens were well tolerated, with minimal adverse effects and no major complications. Given its efficacy, safety, ease of availability, and cost-effectiveness, magnesium sulfate appears to be a better alternative for intraperitoneal instillation during LC. Incorporating intraperitoneal bupivacaine with either magnesium sulfate or dexmedetomidine as part of multimodal analgesia strategies can improve patient outcomes and enhance recovery after laparoscopic surgeries.
